# Statistical limits and conditional complexity in real-world reinforcement learning: a tutorial survey

**DOI:** 10.3389/frai.2026.1847643

**Published:** 2026-06-24

**Authors:** Amar Ahmad, Yvonne Vallès, Youssef Idaghdour

**Affiliations:** Public Health Research Center, NYU Abu Dhabi Research Institute, New York University Abu Dhabi, Abu Dhabi, United Arab Emirates

**Keywords:** deep RL, high dimensionality, Markov decision processes, nonstationarity, partial observability, reinforcement learning, sample complexity, statistical learning theory

## Abstract

Reinforcement learning (RL) has achieved remarkable success in controlled environments, demonstrating superhuman performance in domains such as game playing and simulated robotics. However, its transition to real-world applications remains constrained by fundamental statistical challenges that limit scalability, reliability, and safety. This study systematically examines four such challenges: sample inefficiency, in which millions of interactions may be required even for simple tasks; nonstationarity, arising from evolving environmental dynamics and agent-induced distribution shifts; partial observability, which violates the Markov assumption and inflates estimation variance; and the curse of dimensionality, which causes exploration demands to grow rapidly in high-dimensional spaces. Known theoretical lower bounds from the literature are reviewed to characterize the fundamental limits of these challenges, and a survey of contemporary mitigation strategies is presented, including model-based methods, robust Markov decision process formulations, memory-augmented architectures, and hierarchical abstractions. In addition to these core statistical challenges, this study briefly discusses related deployment-oriented topics—including safe RL, explainable RL, multi-agent coordination, and curriculum learning—that interact with, but remain distinct from, the four primary statistical limits. This study is intended as a tutorial synthesis rather than a source of new theoretical results. Its main contribution is organizational and interpretive: It unifies existing lower bounds, representative complexity arguments, and practical mitigation strategies around the structural assumptions that make real-world RL easier or harder.

## 1 Introduction

Reinforcement learning (RL) is a subfield of machine learning, and more broadly artificial intelligence, that trains agents to make sequential decisions in an environment (Ghasemi and Ebrahimi, 2024). An agent is an entity that perceives its environment and acts to achieve specific aims (Chapman et al., 2023). Learning proceeds by trial-and-error, guided by reward signals (Sutton and Barto, 2018; Kaelbling et al., 1996; Arulkumaran et al., 2017). Unlike supervised learning, which relies on labeled examples, RL must cope with sparse or delayed rewards, leading to challenging credit-assignment problems (Minsky, 1961). For instance, beyond its celebrated game-playing triumphs, RL now optimizes real-time resource allocation in Google data centers, reducing cooling energy consumption by approximately 40% under controlled deployment conditions, as reported by DeepMind in 2016 (Evans and Gao, 2016). Because such figures depend on the baseline, facility, and measurement period, they should be interpreted as application-specific rather than universal evidence of RL performance.

Since 2019, deep reinforcement learning (DRL) has advanced well beyond the original Deep Q-Network (DQN) and AlphaGo era. Algorithms such as MuZero's model-based tree search, population-based actor-critic systems like AlphaStar, and large language model fine-tuning via RL from human feedback (RLHF) have achieved strong performance across multi-agent strategy games, complex continuous control benchmarks, and natural language generation (Schrittwieser et al., 2020; Vinyals et al., 2019; Jaques et al., 2019; Ouyang et al., 2022).

These successes, however, intensify longstanding statistical challenges: state-of-the-art agents often require millions of environment interactions or human-preference labels, substantial computational resources, and may still exhibit instability, limited generalization, and poor interpretability, particularly in safety-sensitive domains such as autonomous driving and surgical robotics (Sutton and Barto, 2018; Dulac-Arnold et al., 2021).

Recent surveys on AI-driven automation present broader taxonomies of intelligent automation systems and discuss challenges related to scalability, reliability, safety, and human–machine integration (Jin et al., 2025; Shi et al., 2026). Compared with these automation-oriented reviews, the present survey focuses more specifically on the statistical limits of RL, including sample complexity, nonstationarity, partial observability, and high dimensionality. Recent multi-agent reinforcement learning (MARL) studies addressing representation-driven sampling, adaptive policy resetting, and offline-to-online transfer are also relevant because they target robustness, exploration, and adaptation in complex environments. However, unlike these algorithm-specific works, this survey emphasizes the underlying statistical problem settings and the conditions under which learning complexity can be reduced.

In this survey, real-world RL refers to RL settings in which data collection is costly or risky, the environment may change over time, observations may be incomplete or noisy, and state-action spaces are often high-dimensional. This includes applications such as robotics, autonomous driving, healthcare, energy systems, and data center control. Thus, the term “real-world” is used here not as a single formal model but as an umbrella term for deployment settings in which sample efficiency, robustness, memory, safety, and scalability are central constraints.

Contributions and scope. This article is intended as a tutorial survey rather than a source of new algorithms or new minimax lower bounds. Its contribution is organizational: it brings together statistical lower-bound intuitions, practical mitigation strategies, and structural assumptions under a common conditional-complexity perspective. In particular, the survey (i) reviews four recurring statistical obstacles in real-world RL, (ii) distinguishes formal results from conceptual synthesis, and (iii) highlights when additional structure, such as stationarity, observability, low intrinsic dimension, or safety constraints, can reduce effective learning complexity.

This paper explores these statistical challenges in detail, synthesizing insights from recent research to provide a structured picture of the obstacles that limit RL's broader applicability. It also surveys state-of-the-art strategies for tackling these obstacles, highlights emerging techniques, and pinpoints open research questions. To make the connection between theory and practice explicit, this survey organizes each major challenge around a common analytical template. For every obstacle, we first state a canonical problem formulation and the associated performance metric under which fundamental lower bounds are known (Section 2.3). Finally, we identify the structural assumptions, such as stationarity, observability, low intrinsic dimension, or model regularity, under which these limits can be relaxed, and relate them to representative algorithmic strategies (Sections 3.1, 3.2, 3.3, and 3.4). This organization is intended to clarify not only what limits RL in worst-case settings, but also how and why additional structure enables improved performance in realistic environments.

## 2 Background

### 2.1 Basic RL formulation

We consider standard RL problems formulated as Markov decision processes (MDPs)


M=(S,A,P,R,γ),

where S and A denote the state and action spaces, *P* is the transition kernel, *R* is the reward function, and γ ∈ [0, 1) is the discount factor. The aim is to find a policy π that maximizes the expected discounted return:


$$\begin{array}{l} \pi^{⋆}=\arg\underset{\pi }{\max}{\text{𝔼}}_{\pi }\left[\overset{\infty }{\underset{t=0}{\sum }}\gamma^{t}R\left(s_{t},a_{t}\right)\right] \end{array}$$ (1)

This formulation provides the baseline against which later deviations—including nonstationarity, partial observability, and high dimensionality—are discussed.

**Figure 1:**
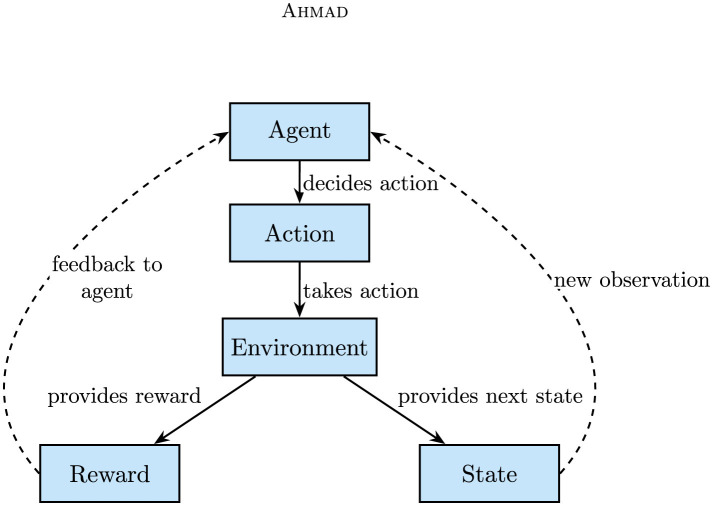
The canonical agent-environment interaction loop in reinforcement learning.

As illustrated in Figure 1, the reinforcement-learning interaction is a closed feedback loop between an agent and its environment. At each discrete time step *t*, the agent observes (or partially observes) a state *s*_*t*_, decides on an action *a*_*t*_, and executes that action in the environment. In response, the environment yields a scalar reward *r*_*t*+1_ that quantifies immediate utility and transitions to the next state *s*_*t*+1_. This reward-state pair constitutes the only feedback available to the agent, which must discover a policy that maximizes the long-term, discounted return defined in Equation 1. Figure 1 makes explicit the dual channels of feedback, reward, and state, and highlights both the forward action path and the back-propagating information flow that drives learning.

### 2.2 Deep reinforcement learning

**Figure 2:**
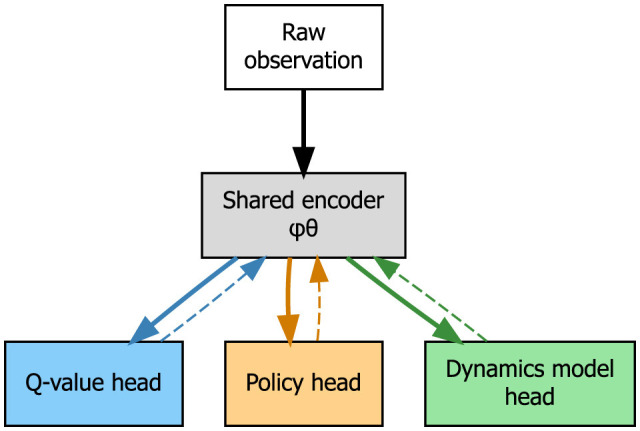
Canonical deep reinforcement learning architecture. A shared encoder processes observations, while separate heads implement value-based, policy-based, and model-based objectives.

Deep reinforcement learning (DRL) augments classical RL with the representation power of deep neural networks and has become the de facto approach whenever raw, high-dimensional inputs (images, LiDAR scans, natural language) must be mapped to actions or value estimates (Goodfellow et al., 2016; Ahmed et al., 2025; Mnih et al., 2015; Silver et al., 2017). Figure 2 illustrates the three algorithmic pillars that now form the DRL toolbox: value-based, policy-based, and model-based methods.

#### 2.2.1 Value-based learning

A network $Q_{\theta }:S\times A\to \mathbb{R}$ approximates the action-value function. The DQN loss function


$$\begin{array}{l} L_{\text{DQN}}\left(\theta \right)={\text{𝔼}}_{\left(s,a,r,s^{\prime }\right)}\left[{\left(r+\gamma \underset{a^{\prime }}{\max}Q_{\theta^{-}}\left(s^{\prime },a^{\prime }\right)-Q_{\theta }\left(s,a\right)\right)}^{2}\right] \end{array}$$ (2)

is minimized by stochastic gradient descent; θ^−^ denotes a target network updated every *K* steps to break the deadly triad (bootstrapping + off-policy + function approximation) that otherwise leads to divergence. Here, one forces the network *Q*_θ_ to predict a target that is (temporarily) treated as the ground truth; as training proceeds, the target network is refreshed, slowly propagating more accurate value estimates throughout the entire state-action space.

#### 2.2.2 Policy-gradient learning

A stochastic policy π_θ_(*a*∣*s*) with parameters θ ∈ ℝ^*d*^ is optimized by ascending an unbiased estimate of the performance gradient


$$\begin{array}{l} \nabla_{\theta }J\left(\theta \right)={\text{𝔼}}_{s\sim d_{\pi_{\theta }},a\sim \pi_{\theta }}\left[\nabla_{\theta }\log\pi_{\theta }\left(a|s\right)A_{\pi_{\theta }}\left(s,a\right)\right], \end{array}$$ (3)

where *d*_π_θ__(*s*) is the discounted state-visitation distribution and *A*_π_θ__ is any advantage estimator. Choosing *A*_π_θ__(*s, a*) = *Q*_π_θ__(*s, a*)−*V*_π_θ__(*s*) yields the classical variance-reduced actor-critic form.


$$Q_{\pi_{\theta }}\left(s,a\right)={\text{𝔼}}_{\pi_{\theta }}\left[\overset{\infty }{\underset{t=0}{\sum }}\gamma^{t}R_{t}|S_{0}=s,A_{0}=a\right],$$

$$V_{\pi_{\theta }}\left(s\right)={\text{𝔼}}_{\pi_{\theta }}\left[\overset{\infty }{\underset{t=0}{\sum }}\gamma^{t}R_{t}|S_{0}=s\right].$$

The action-value function *Q*_π_θ__ returns the expected discounted return obtained by taking action *a* in state *s* once and following π_θ_ thereafter. The state-value function *V*_π_θ__ is the same expectation when the first action is also selected according to π_θ_. Their difference


$$A_{\pi_{\theta }}\left(s,a\right)=Q_{\pi_{\theta }}\left(s,a\right)-V_{\pi_{\theta }}\left(s\right)$$

quantifies how much better (positive advantage) or worse (negative advantage) action *a* is relative to the policy's average behavior at state *s*. Substituting this definition into Equation 3 yields the baseline-corrected policy-gradient estimator used in modern actor-critic algorithms such as A2C, PPO, and TRPO.

##### 2.2.2.1 Known Result 1 (baseline variance reduction)

Let $g\left(a\right)=\nabla_{\theta }\log\pi_{\theta }\left(a|s\right)\in {\mathbb{R}}^{d}$ and let *b*(*s*) ∈ ℝ be any baseline that is *independent* of the action *a*. Then


$$V_{a\sim \pi_{\theta }}\left[g\left(a\right)\left(Q\left(s,a\right)-b\left(s\right)\right)\right]=V_{a\sim \pi_{\theta }}\left[g\left(a\right)Q\left(s,a\right)\right]$$

$$-||{\text{𝔼}}_{a\sim \pi_{\theta }}\left[g\left(a\right)\left(Q\left(s,a\right)-b\left(s\right)\right)\right]{||}_{2}^{2},$$ (4)

and the variance is minimized when


$$b^{⋆}\left(s\right)=V_{\pi_{\theta }}\left(s\right)={\text{𝔼}}_{a\sim \pi_{\theta }}\left[Q\left(s,a\right)\right].$$

Hence, the optimal baseline does *not* change the expected policy gradient but strictly reduces its variance unless *Q*(*s*, ·) is constant.

*Proof sketch*. Throughout the proof, the expectation *E*[·] is understood to be over *a*~π_θ_(·∣*s*); one suppresses the conditioning on *s* to keep formulas short.

##### 2.2.2.2 Step 1

Because *b*(*s*) is a constant with respect to the integration over *a* and, owing to the *score-function identity* *E*[*g*(*a*)] = ∇_θ_∫π_θ_(*a*∣*s*)*da* = 0, one has


𝔼[g(a)(Q-b)]=𝔼[g(a)Q]-b𝔼[g(a)]=𝔼[g(a)Q].

Thus, adding or removing *b*(*s*) does not bias the gradient estimator.

##### 2.2.2.3 Step 2

For a vector random variable *X* one uses $V\left[X\right]=\text{𝔼}\left[||X{||}_{2}^{2}\right]-||\text{𝔼}\left[X\right]{||}_{2}^{2}$. Setting *X* = *g*(*Q*−*b*) yields


$$\mathrm{Var}\left[g\left(Q-b\right)\right]=\text{𝔼}\left[g_{2}^{2}{\left(Q-b\right)}^{2}\right]-{\text{𝔼}\left[g\left(Q-b\right)\right]}_{2}^{2}.$$

Because the second term is independent of *b* (Step 1), Equation 1 follows immediately.

##### 2.2.2.4 Step 3

Define *f*(*b*): = *E*[||*g*||^2^(*Q* − *b*)^2^]. Since the baseline does not change the mean gradient, minimizing the variance is equivalent to minimizing *f*(*b*). Differentiating,


$$\frac{df}{db}=\text{𝔼}\left[||g{||}^{2}2\left(b-Q\right)\right]=2\left(b\text{𝔼}\left[||g{||}^{2}\right]-\text{𝔼}\left[||g{||}^{2}Q\right]\right).$$

Setting the derivative to zero gives


$$b^{⋆}\left(s\right)=\frac{\text{𝔼}\left[||g\left(a\right){||}^{2}Q\left(s,a\right)\right]}{\text{𝔼}\left[||g\left(a\right){||}^{2}\right]}.$$ (5)

In special cases where ||*g*(*a*)||^2^ is constant across actions, this reduces to $b^{⋆}\left(s\right)=V_{\pi_{\theta }}\left(s\right)$.

##### 2.2.2.5 Step 4

For the tabular case, or any softmax policy whose logits are linear in θ, ||*g*(*a*)||^2^ is the same for every action *a* taken in state *s*; it can therefore be pulled out of both expectations in Equation 5, canceling top and bottom and leaving


$$b^{⋆}\left(s\right)=\text{𝔼}\left[Q\left(s,a\right)\right]=V_{\pi_{\theta }}\left(s\right).$$

##### 2.2.2.6 Step 5

Unless *Q*(*s, a*) is constant in *a*, the random variable *Q*−*b*^⋆^(*s*) has non-zero variance, making the subtraction term in Equation 4 strictly positive and the overall variance strictly smaller.

This result summarizes a standard variance-reduction principle in policy-gradient RL. Subtracting a baseline that depends only on the current state does not change the expected policy gradient estimate, but it can substantially reduce its variance. Standard results show that the minimum variance is achieved when the baseline equals the state value function,


$$V_{\pi }\left(s\right)={\text{𝔼}}_{a\sim \pi }\left[Q\left(s,a\right)\right],$$

which improves gradient stability whenever actions within a state have different values.

#### 2.2.3 Actor-critic implementation

Actor-critic algorithms operationalize this variance-reduction principle by jointly learning a state-value estimator ${\hat{V}}_{\psi }\left(s\right)$ together with the policy π_θ_. At each step, they


$$\begin{array}{l} \text{critic update:}\psi \leftarrow \psi -\eta_{v}\nabla_{\psi }{\left(r_{t}+\gamma {\hat{V}}_{\psi }(s_{t+1})-{\hat{V}}_{\psi }(s_{t})\right)}^{2},\\ \text{actor update: }\theta \leftarrow \theta +\eta_{\pi }{\hat{A}}_{t}\nabla_{\theta }\log\pi_{\theta }(a_{t}\text{​}|\text{​}s_{t}),] \end{array}$$

$${Â}_{t}=r_{t}+\gamma {\hat{V}}_{\psi }\left(s_{t+1}\right)-{\hat{V}}_{\psi }\left(s_{t}\right).$$

Sharing an encoder between π_θ_ and ${\hat{V}}_{\psi }$ provides rich gradients for representation learning, while separate learning rates η_π_, η_*v*_ allow practitioners to balance bias and variance. Many modern variants add an entropy bonus $-\beta \underset{a}{\sum }\pi_{\theta }\left(a\right)\log\pi_{\theta }\left(a\right)$ to encourage exploration and further stabilize updates.

#### 2.2.4 Model-based learning

DRL can learn an approximate dynamics model ${\hat{P}}_{\eta }\left(s^{\prime }|s,a\right)$ and reward ${\hat{R}}_{\eta }\left(s,a\right)$, enabling imagined roll-outs that greatly improve sample efficiency (e.g. MuZero; Schrittwieser et al., 2020). The price is model bias: planning with an inaccurate ${\hat{P}}_{\eta }$ can degrade performance. Hybrid approaches trade off real and synthetic data via uncertainty-aware rollout schedules.

#### 2.2.5 Practical challenges

The expressive power of deep networks comes with three statistical headaches: (i) **Instability:** Bootstrapping through noisy targets can amplify estimation error, causing oscillations or divergence. (ii) **Catastrophic forgetting:** Online updates overwrite earlier knowledge; experience replay or elastic weight consolidation alleviate but do not eliminate the problem. (iii) **Data hunger:** High-dimensional encoders often contain millions of parameters; even with modern data-efficiency tricks, DRL typically consumes 10^6^-10^9^ transitions-orders of magnitude above human experience.

State-of-the-art research therefore focuses on (i) distributional value functions, (ii) contrastive or self-supervised auxiliary losses that shape latent representations, and (iii) scalable transformers with sequence-level credit assignment, all aiming to stabilize training and squeeze more generalization out of each environment interaction (Kapturowski et al., 2019; Schwarzer et al., 2021).

#### 2.2.6 Illustrative synthetic simulation

To visualize how sample efficiency and instability affect different algorithms, an illustrative synthetic simulation study was conducted in which learning curves were generated from a calibrated probabilistic model rather than from actual Atari training runs.

**Figure 3:**
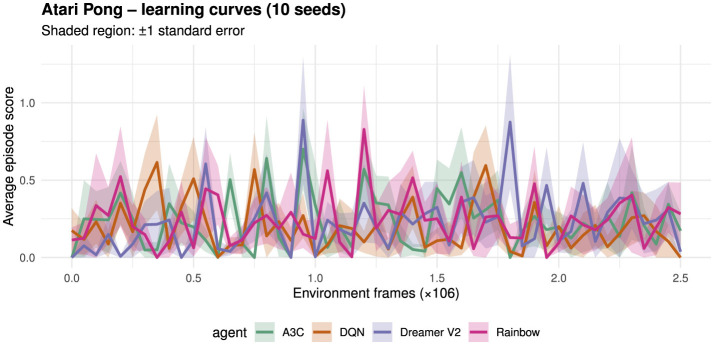
Synthetic illustrative Atari Pong learning curves generated from a simulated probabilistic model rather than empirical Atari experiments. Solid lines represent simulated mean episode return across 10 random seeds, while shaded regions denote ±1 simulated standard error. The figure is intended only to illustrate qualitative differences in sample efficiency and training instability across RL algorithms.

Figure 3 presents synthetic illustrative learning curves and should not be interpreted as empirical Atari benchmark results. The curves were generated using a calibrated probabilistic simulation framework designed solely to visualize qualitative differences in learning speed, variability, and instability across algorithms.

Figure 3 compares synthetic learning curves for four DRL agents applied to Atari Pong: DQN, Rainbow, A3C, and Dreamer V2, each averaged over ten simulated training runs. The curves were generated from a calibrated probabilistic model rather than from actual experiments. For each agent, an asymptotic performance ceiling, a characteristic learning-rate parameter, a stochastic noise component, and a small probability of instability events that produce temporary performance drops were specified. Overall improvement was modeled using a sigmoid-type growth curve with additive Gaussian noise, whereas occasional negative spikes were introduced to emulate divergence or catastrophic updates commonly observed in DRL practice.

Because the curves are synthetic, Figure 3 should be interpreted only as a pedagogical visualization rather than evidence about the empirical ranking of algorithms. The figure illustrates qualitative phenomena often reported in DRL studies: different algorithms may exhibit varying apparent learning rates, varying levels of run-to-run variability, and occasional instability. In this illustrative setup, the model-based and enhanced value-based curves are assigned faster improvement and lower variance than the vanilla baselines. These choices are intended to make the statistical issues visually concrete rather than to provide benchmark conclusions about Atari Pong or about the relative superiority of one algorithm over another.

### 2.3 Problem statements and evaluation metrics

To ground the subsequent discussion of statistical limits and conditional complexity, we briefly formalize the canonical problem classes and evaluation metrics that recur throughout modern RL theory. Our aim here is not to introduce new models, but to make explicit the problem formulations with respect to which lower bounds, impossibility results, and structural trade-offs are typically stated.

#### 2.3.1 Episodic and continuing MDP learning

The standard RL problem is defined over a Markov decision process (MDP) (S,A,P,R,γ), where the agent seeks a policy π maximizing expected discounted return


$$J\left(\pi \right)={\text{𝔼}}_{\pi }\left[\overset{\infty }{\underset{t=0}{\sum }}\gamma^{t}R\left(s_{t},a_{t}\right)\right].$$

Performance is commonly measured either by cumulative regret over *T* interactions,


$$\text{Regret}\left(T\right)=\overset{T}{\underset{t=1}{\sum }}\left(V^{\pi^{⋆}}\left(s_{t}\right)-V^{\pi_{t}}\left(s_{t}\right)\right),$$

or by sample complexity, defined as the number of interactions required to identify an ε-optimal policy with high probability. These metrics underpin most worst-case lower bounds for tabular and function-approximation settings.

#### 2.3.2 Nonstationary and piecewise-stationary environments

In real-world settings, transition dynamics and rewards may evolve over time. A common abstraction is the piecewise-stationary MDP, in which the environment switches among a finite sequence of stationary MDPs. Performance is then evaluated in terms of dynamic regret or adaptation delay, often parameterized by the number of changepoints or a variation budget measuring cumulative drift in the transition kernel. Such formulations make explicit the trade-off between stability assumptions and achievable learning guarantees.

#### 2.3.3 Partial observability and memory constraints

In partially observable MDPs (POMDPs), the agent observes *o*_*t*_~*O*(·∣*s*_*t*_) rather than the latent state *s*_*t*_. The optimal policy may depend on the full history *h*_*t*_ = (*o*_1_, *a*_1_, …, *o*_*t*_). Problem complexity is therefore measured not only by regret or sample efficiency but also by the memory or belief-state dimension required to approximate optimal behavior. Lower bounds in this setting typically characterize the growth of memory requirements as a function of the horizon, the state space size, or the observation ambiguity.

#### 2.3.4 High-dimensional and structured state spaces

When S is large or continuous, performance guarantees depend on additional structural assumptions, such as Lipschitz continuity, low intrinsic dimension, or linear representability. Evaluation metrics in this regime often scale with covering numbers, effective dimension, or model complexity measures rather than raw state cardinality. These formulations clarify how representational structure mediates the curse of dimensionality.

#### 2.3.5 Robustness and safety criteria

Finally, in safety-critical or misspecified environments, agents are evaluated using worst-case or risk-sensitive objectives. Examples include minimax regret over uncertainty sets of transition kernels, conditional value-at-risk (CVaR) objectives, or constrained formulations that penalize unsafe states. These metrics explicitly encode robustness requirements that fundamentally alter achievable statistical guarantees.

Taken together, these problem formulations and metrics provide the reference frame for the statistical limits discussed in the remainder of the paper. In subsequent sections, we examine how different structural assumptions selectively relax these limits, giving rise to the conditional complexity regimes that characterize real-world RL.

## 3 Major statistical challenges in RL

### 3.1 Sample inefficiency

Deep reinforcement learning (DRL) algorithms often require extensive exploration to achieve high performance, making them computationally expensive and time-consuming (Schaul et al., 2016). This issue is exacerbated in real-world tasks where data scarcity is a significant concern. While simulations can generate unlimited data, real-world data collection remains costly and resource-intensive. Furthermore, long-horizon credit assignment is a persistent challenge; delayed rewards make it difficult to determine which actions contributed to success or failure.

The representative bounds, known results, and illustrative propositions in this section summarize standard lower-bound constructions and complexity arguments from the RL and bandit literature. They are presented in a simplified and unified form for pedagogical purposes rather than as new theoretical contributions.

#### 3.1.1 Representative Bound 1 (tabular sample-complexity lower bound).

Consider any finite MDP with state set S, action set A, and discount factor γ ∈ (0, 1). Fix accuracy ε ∈ (0, 1) and confidence 1−δ. Any RL algorithm that, with probability at least 1−δ, outputs a *Q*-function $\hat{Q}$ satisfying


$$\|\hat{Q}-Q^{⋆}{\|}_{\infty }\leq \varepsilon$$

must collect at least


$$\text{Ω}\left(\frac{\left|S||A\right|}{\varepsilon^{2}{\left(1-\gamma \right)}^{3}}\log\frac{1}{\delta }\right)$$

sample transitions in the worst case.

*Proof sketch*. Reduce the RL task to pure best arm identification.

**Step 1:** Construct an MDP $M_{\left(\mu_{1},\ldots ,\mu_{K}\right)}$ with K=|S||A| disjoint state-action pairs. For every pair *k*: = (*s*_*k*_, *a*_*k*_):

- Executing *a*_*k*_ in *s*_*k*_ yields an i.i.d. reward *R*_*k*_~Bernoulli(μ_*k*_),
- then deterministically returns to *s*_*k*_ (self-loop).

All other transitions are impossible, so each pair behaves like an independent bandit arm. Let $\mu^{⋆}:=\underset{k}{\max}\mu_{k}$ and choose the vector (μ_1_, …, μ_*K*_) so that exactly one arm is optimal and $|\mu^{⋆}-\mu_{k}|=\varepsilon \left(1-\gamma \right)$ for every *k*≠*k*^⋆^.

**Step 2:** Because the process remains in *s*_*k*_ forever,


$$Q^{⋆}\left(s_{k},a_{k}\right)=\frac{\mu_{k}}{1-\gamma }\;\text{and }V^{⋆}\left(s_{k}\right)=\frac{\mu_{k}}{1-\gamma }.$$

Thus distinguishing the ε-optimal *Q*-function is equivalent to identifying the best arm up to Δ = ε(1−γ) in the bandit instance (μ_1_, …, μ_*K*_).

**Step 3:** For pure exploration, any algorithm that returns an arm whose mean is within Δ of μ^⋆^ with probability ≥1 − δ must draw, in the worst case, at least


$$\text{Ω}\left(\frac{K}{{\text{Δ}}^{2}}\log\frac{1}{\delta }\right)=\text{Ω}\left(\frac{\left|S||A\right|}{\varepsilon^{2}{\left(1-\gamma \right)}^{2}}\log\frac{1}{\delta }\right)$$

samples (Mannor and Tsitsiklis, 2004, Thm. 3).

**Step 4:** Each sample of the bandit arm corresponds to one environment transition in the MDP, so the same bound applies to any RL algorithm. Finally, standard bias-variance arguments (e.g. Sutton and Barto, 2018, Eq. 6.6) show that estimating *Q*^⋆^ within ε additionally costs a factor (1−γ)^−1^, yielding the claimed


$$\text{Ω}\left(\frac{\left|S||A\right|}{\varepsilon^{2}{\left(1-\gamma \right)}^{3}}\log\frac{1}{\delta }\right)$$

interactions.

Hence no reinforcement-learning algorithm can guarantee $||\hat{Q}-Q^{⋆}{||}_{\infty }\leq \varepsilon$ with confidence 1−δ using fewer samples.

Representative Bound 3.1.1 summarizes a standard sample-complexity lower bound for finite Markov decision processes. In the worst case, any RL algorithm that aims to estimate the optimal action-value function *Q*^⋆^ uniformly within ε with probability at least 1−δ requires on the order of


$$\frac{\left|S||A\right|}{\varepsilon^{2}{\left(1-\gamma \right)}^{3}}\log\frac{1}{\delta }$$

environment transitions. Thus, sample complexity scales linearly with the number of state-action pairs, quadratically with the desired accuracy, cubically with the discount factor as it approaches 1, and logarithmically with the confidence level.

**Corollary 1**. *Fix accuracy ε* ∈ (0, 1), *confidence* 1−δ, *and discount* γ ∈ (0, 1). *For every reinforcement-learning algorithm* A *there exists a finite MDP on which* A *must collect at least*


$$\text{Ω}\left(\frac{\left|S||A\right|}{\varepsilon^{2}{\left(1-\gamma \right)}^{3}}\log\frac{1}{\delta }\right)$$

*environment transitions before it can guarantee* $∥\hat{Q}-Q^{⋆}{∥}_{\infty }\leq \varepsilon$ *with probability* ≥1−δ. *Conversely, existing model-based algorithms such as E^3^* (Kearns and Singh, 2002), *R-Max (Brafman and Tennenholtz, 2002),* *and UCB-VI (2017Azar et al.,) achieve*


$$O\left(\frac{\left|S||A\right|}{\varepsilon^{2}{\left(1-\gamma \right)}^{3}}\text{polylog}\left(\frac{\left|S||A\right|}{\delta }\right)\right)$$

*samples. Hence, the minimax sample complexity for tabular discounted RL is*


$$\Theta \left(\frac{\left|S||A\right|}{\varepsilon^{2}{\left(1-\gamma \right)}^{3}}\log\frac{1}{\delta }\right).$$

Corollary 1 establishes that, for any learning method, there exists a finite MDP under which at least on the order of


$$\frac{\left|S||A\right|}{\varepsilon^{2}{\left(1-\gamma \right)}^{3}}\log\frac{1}{\delta }$$

interaction steps are required before an ε-accurate estimate of the optimal *Q*-values can be obtained with high confidence. Moreover, well-known model-based algorithms are shown to achieve this bound up to logarithmic factors, implying that this rate is minimax-optimal in the tabular discounted setting.

These methods reduce sample requirements only under favorable assumptions. Experience replay can introduce bias when the replay distribution differs substantially from the current policy distribution. Model-based rollouts are useful only when model error is controlled; otherwise, synthetic data can amplify bias. Transfer and meta-RL can improve adaptation, but may fail under task mismatch or distribution shift. Thus, sample-efficiency gains are conditional rather than universal.

#### 3.1.2 Mitigation strategies

Several approaches have been proposed to address sample inefficiency. Off-policy learning methods, such as experience replay, allow agents to reuse past experiences for learning. Model-based RL generates synthetic rollouts using learned models of the environment, significantly reducing the need for real-world data (Chua et al., 2018). Furthermore, transfer learning and meta-reinforcement learning (Meta-RL) enable knowledge reuse across tasks, improving efficiency in new environments (Finn et al., 2017).

### 3.2 Nonstationarity

RL agents often encounter nonstationary environments, where the underlying dynamics evolve, rendering previously learned policies ineffective (Dietterichc, 2000). This challenge stems from two primary sources. First, dynamics change when the environment's transition probabilities (*P*) or reward functions (*R*) shift unexpectedly. Second, agent-induced drift arises as the agent's policy improves, altering the data distribution it encounters during exploration. These forms of nonstationarity complicate learning and require adaptive mechanisms to maintain performance.

Table 1 summarizes common approaches for handling nonstationarity in reinforcement learning.

**Table 1:** Common approaches for handling nonstationarity in reinforcement learning.

Method family	Main idea	Strength	Limitation
Sliding-window methods	Use recent data only	Simple adaptation to drift	May discard useful old data
Restart/change-point methods	Detect regime shifts and restart learning	Good for abrupt changes	Detection can be delayed or noisy
Robust MDP methods	Optimize against uncertainty sets	Stable under model uncertainty	Often conservative
Meta-RL/adaptive policies	Learn to adapt quickly across tasks	Useful for recurring changes	Requires diverse training tasks
Continual learning	Preserve and update knowledge over time	Reduces forgetting	Stability–plasticity trade-off

Therefore, nonstationarity is not solved by a single algorithmic principle. Abrupt regime changes, slow drift, and agent-induced distribution shift require different assumptions and different adaptation mechanisms.

These approaches also involve trade-offs. Continual-learning methods must balance plasticity against catastrophic forgetting, robust MDP methods can be overly conservative, and ensembles increase computational cost without guaranteeing fast adaptation to abrupt regime shifts. Consequently, successful handling of nonstationarity depends strongly on whether changes are gradual, abrupt, recurring, or adversarial.

Supplementary Figure S1 illustrates the challenges RL agents face when operating in nonstationary environments, as well as the mechanisms required to address these challenges. At the top of the diagram, the RL agent interacts with a nonstationary environment, which introduces two primary sources of difficulty. The first is Changing Dynamics, where the environment's transition probabilities or reward functions shift unexpectedly, creating instability in the agent's learning process. The second challenge is Agent-Induced Drift, which arises as the agent's policy evolves, altering the data distribution it encounters during exploration and learning.

These two sources of nonstationarity lead to significant issues, such as training instability and performance degradation, which can severely hinder the agent's ability to learn effective policies. Addressing these problems requires implementing adaptive mechanisms, such as robust Markov decision process formulations and continual learning methods, that enable the agent to adapt to changing environmental dynamics and sustain its performance over time.

The RL agent is depicted as encountering changing dynamics while simultaneously introducing agent-induced drift into the environment. Both factors directly contribute to training and performance challenges. These challenges, in turn, necessitate the development and use of adaptive mechanisms to ensure stability and effectiveness in learning.

Supplementary Figure S1 provides a structured and intuitive visualization of how nonstationary environments pose challenges to RL agents. It also emphasizes the importance of designing mechanisms that enable agents to overcome these challenges and thrive in dynamic, evolving environments.

From Nonstationarity to Adaptive Guarantees **Problem class**. Piecewise-stationary MDPs with unknown changepoints and a bounded variation budget *V*_*T*_ on the transition dynamics.

**Canonical limitation**. In the absence of stationarity, static regret bounds scale linearly with *T*, and no sublinear regret is achievable without additional assumptions.

**Representative bound**. For *K* changepoints, minimax dynamic regret typically scales as

$$\text{Ω}\left(\sqrt{KT}\right),$$

even in tabular settings.

**Mechanism enabling improvement**. Adaptive windowing, restart strategies, or change-point detection allow policies to reset or reweight past data.

**Structural assumption required**. Bounded variation or sparsely occurring regime changes. Under these conditions, learning guarantees that it interpolates smoothly between stationary and fully adversarial regimes.

#### 3.2.1 Mitigation strategies

Addressing nonstationarity involves approaches such as continual or lifelong learning, which enable agents to adapt incrementally as environmental dynamics change (Ring, 1994). Another effective approach is the use of robust MDP formulations, which account for uncertainties in transitions and rewards, ensuring more reliable decision-making (Nilim and El Ghaoui, 2005). Additionally, ensemble methods can hedge against variance by combining predictions from multiple models, helping agents remain robust in dynamic settings.

### 3.3 Partial observability

In many real-world scenarios, agents operate under partial observability, where only incomplete or noisy observations of the environment are available. This violates the Markov assumption, which holds that the current state contains all necessary information for decision-making. Hidden states and sensor noise exacerbate this challenge, making it difficult for agents to infer the true state of the environment and take optimal actions.

#### 3.3.1 Representative Bound 3 (linear memory requirements under partial observability)

For every horizon *H*≥1 there exists a finite episodic POMDP $P_{H}$ such that

1. The optimal value is *V*^⋆^ = *H*, attainable by a policy that stores the entire sequence of past observations.
2. Any policy whose internal state (memory) is represented by at most *m*<*H* bits is bounded away from the optimal return *H*.

Hence, Θ(*H*) bits of memory are necessary and sufficient for optimality in the worst case.

*Proof sketch*. Fix *H* and construct the POMDP $P_{H}$.

At the beginning of each episode, Nature samples a binary string $\theta =\left(\theta_{1},\ldots ,\theta_{H}\right)\in {\left\{0,1\right\}}^{H}$ uniformly at random. At decision time *t* ∈ {1, …, *H*} the true (unobserved) state is (*t*, θ).

The agent can choose


OBSERVE, REPORT0, REPORT1.

When the agent plays Observe at step *t* it receives the symbol θ_*t*_ ∈ {0, 1} and reward 0. If instead it plays Report(*b*) with *b* ∈ {0, 1} it receives a dummy observation ⊥ and reward


$$r_{t}=1\left\{b=\theta_{t}\right\}.$$

After either action, the environment deterministically advances to time *t*+1. The episode terminates after step *H*.

A controller that retains the entire observation history executes:


$$\text{at each}t:\;\text{OBSERVE}\left(\text{store}\theta_{t}\right),\text{REPORT}\left(\theta_{t}\right)\text{at}t+1.$$

Because the report for θ_*t*_ is delivered one step later, remembering a single bit for each time index is sufficient and necessary. This policy earns a reward of 1 at every step, so *V*^⋆^ = *H*.

Let a controller have at most *m*<*H* bits of internal state, meaning its internal variable can assume at most 2^*m*^ distinct values over the course of an episode. Any finite-state controller, bounded-depth recurrent neural network (RNN), or finite-precision RAM can be represented within this framework.

Correctly reporting θ_*t*_ requires retaining information about the corresponding hidden bit until the time of reporting. Therefore, a controller that is correct on many distinct indices must preserve the relevant bits in its internal memory. If the controller has only *m*<*H* bits of effective memory, then at most *m* independent bits can be stored without loss in the worst case. For the remaining *H*−*m* indices, the controller cannot deterministically recover the corresponding hidden bits from memory alone and, under the symmetric construction above, can do no better than guessing, succeeding with probability 1/2 on each such index. Consequently,


$$\text{𝔼}\left[R\right]\leq m\times 1+\left(H-m\right)\times \frac{1}{2}=\frac{H+m}{2}<H,$$

for every *m*<*H*.

Thus, the expected return is bounded away from the optimal value *H* whenever the memory capacity is strictly smaller than the horizon. In particular, this simplified construction shows that insufficient memory produces a linear performance gap of at least


$$H-\frac{H+m}{2}=\frac{H-m}{2}.$$

The case *m* = 0 corresponds to a memoryless controller, for which the same argument gives an expected return at most *H*/2 under random guessing. Stronger adversarial constructions can force even larger gaps for memoryless policies, but the present example is intended only to illustrate the basic linear dependence of memory requirements on the horizon.

Representative Bound 3.3.1 highlights a central limitation in partially observable control: memory itself can become the dominant source of complexity, independent of exploration or optimization. In the constructed class of tasks, the agent must retain a sequence of one-bit observations long enough to make correct future decisions. Standard information-theoretic arguments imply that any controller with fewer than *H* bits of effective memory, or equivalently fewer than 2^*H*^ latent states, cannot guarantee near-optimal performance in the worst case. Two immediate consequences follow from this observation.

In practical DRL systems, this memory requirement motivates the use of recurrent policies, transformers, and belief-state estimators. RNNs and LSTMs compress observation histories into hidden states, while transformer-based agents retain longer contexts through attention. Belief-state methods instead maintain an explicit probabilistic estimate of the latent state. These approaches do not remove the worst-case memory barrier, but they exploit structure in real environments where only a compressed summary of the history may be sufficient.

**Corollary 2**. *Feed-forward policies, such as multilayer perceptrons (MLPs) that map the current observation directly to an action, correspond to the case *m* = 0 and therefore achieve zero reward on the task family $P_{H}$. This result implies that any expressive *stateless* policy class is fundamentally inadequate in the worst case. To achieve meaningful performance, policies must incorporate some form of internal state, such as recurrent neural networks (RNNs), attention mechanisms with positional embeddings, external memory modules, or belief-state filters*.

**Corollary 3**. *The gap between the optimal return *H* and the best return achievable using an *m*-bit memory (which cannot exceed *m*) grows linearly with the task horizon. Consequently, memory requirements increase proportionally with task length and do not stabilize over time. Effective performance is achievable only when additional structure is present in the environment, such as Markovian dynamics, forgettable sub-tasks, or compressible sufficient statistics. In practical applications, this observation helps explain why fixed-size recurrent models often struggle on long-horizon tasks and motivates the use of adaptive-capacity mechanisms, such as transformers with expanding context windows or models equipped with external memory buffers*.

While the construction is deliberately adversarial, it sets a worst-case benchmark: Θ(*H*) bits of memory are *both* necessary and sufficient for optimal control when no further structure is available. Any attempt to reduce the memory footprint of RL agents must therefore rely on exploiting additional assumptions about the environment, e.g., probabilistic sparsity, predictable sub-aims, or low-rank belief dynamics, rather than generic algorithmic ingenuity alone.

However, memory-based methods do not remove the underlying statistical difficulty. Recurrent networks and transformers can approximate useful history summaries, but may still fail when the required information exceeds their effective context or capacity. Belief-state methods are principled, but depend on accurate observation and transition models. Hence, partial observability is mitigated mainly when the latent state is compressible or inferable from a limited history.

#### 3.3.2 Mitigation strategies

To address partial observability, recurrent architectures such as Recurrent Neural Networks (RNNs) and Long Short-Term Memory (LSTMs) networks are commonly used. These models can process temporal sequences of observations, allowing agents to infer hidden states over time (Hausknecht and Stone, 2015). Another promising approach is Bayesian reinforcement learning, which tracks belief states to maintain a probabilistic estimate of hidden variables (Ghavamzadeh et al., 2016). Offline filtering or smoothing techniques can also be applied to reduce the impact of sensor noise, improving the quality of observations and subsequent decision-making.

From Partial Observability to Memory Requirements **Problem class**. Partially observable MDPs in which the optimal policy depends on the full observation history.

**Canonical limitation**. Any finite-memory policy may incur linear regret when observation ambiguity cannot be resolved from bounded histories.

**Representative bound**. Lower bounds show that memory requirements either grow exponentially with the horizon or linearly with the number of latent states in worst-case POMDPs.

**Mechanism enabling improvement**. Belief-state compression, predictive state representations, or recurrent architectures that approximate sufficient statistics.

**Structural assumption required**. Finite-rank observation models, weak observability, or fast mixing. These assumptions permit bounded-memory approximations without exponential blow-up.

### 3.4 Curse of dimensionality

As the dimensionality of the state or action space increases, RL faces the curse of dimensionality, where large state spaces slow learning and introduce significant variability. Sparse rewards, already challenging in smaller spaces, become even rarer in high-dimensional environments, making it difficult for agents to learn effective policies. Furthermore, deep neural networks used in DRL often require millions of parameters, further complicating training and increasing computational demands.

#### 3.4.1 Representative Bound 2 (minimax lower bound under Lipschitz smoothness)

Let $S={\left[0,1\right]}^{d}$ and A={0,1}. Fix a discount γ ∈ (0, 1) and a Lipschitz constant *L*>0. Denote by M(L) the class of discounted MDPs whose reward function *r* and transition kernel *P* are *L*-Lipschitz in the state:


$$\begin{array}{c} ||P\left(\cdot |s,a\right)-P\left(\cdot |s^{\prime },a\right){||}_{\text{TV}}+|r\left(s,a\right)-r\left(s^{\prime },a\right)|\leq L||s-s^{\prime }{||}_{\infty },\\ \forall s,s^{\prime }\in S,a\in A. \end{array}$$

Suppose an RL algorithm outputs, with probability at least $1-\delta \left(0<\delta <\frac{1}{2}\right)$, a value estimate $\hat{V}$ that satisfies $||\hat{V}-V^{⋆}{||}_{\infty }\leq \varepsilon \left(0<\varepsilon <1\right)$ for *every* MDP in M(L). Then its *expected* number of environment interactions *T* obeys


$$T=\text{Ω}\left({\left(1-\gamma \right)}^{-1}\varepsilon^{-d}\log\frac{1}{\delta }\right).$$

*Proof sketch*. The argument follows the usual information-theoretic route: (i) build a finite family of hard MDPs that are mutually ε-separated in value yet *L*-Lipschitz, (ii) show that producing a uniformly ε-accurate estimate requires identifying the correct member of the family, and (iii) invoke Fano's inequality to lower-bound the sample size.

##### 3.4.1.1 A packing of the state space

Pick the side length $h=\frac{\varepsilon }{4L}$ and grid-partition [0, 1]^*d*^ into cubes *C*_1_, …, *C*_*N*_ of side *h*. Because the cubes are disjoint and have diameter $\leq h\sqrt{d}$, their centers *x*_1_, …, *x*_*N*_ form a *cε*-packing of [0, 1]^*d*^ with *N* = Θ(ε^−*d*^) points.

##### 3.4.1.2 Base MDP $M_{0}$

The transition kernel is *P*_0_(·∣*s, a*) = δ_*s*_, meaning the next state is deterministically the current state. The reward function is identically zero, *r*_0_(*s, a*) = 0 for every state-action pair. Because the agent can neither change the state nor collect any positive reward, every policy produces the same return. The optimal value function is therefore $V_{0}^{⋆}\left(s\right)=0$ for all s∈S; in this trivial MDP, every policy is optimal.

##### 3.4.1.3 A bump-reward construction

Fix a *base* MDP $M_{0}$: **Transition**: *P*_0_(·∣*s, a*) = δ_*s*_ (the state never changes). **Reward**: *r*_0_(*s, a*) = 0 for every (*s, a*). Hence $V_{0}^{⋆}\left(s\right)=0$ for all *s*. For every cube index *i* ∈ [*N*], build a variant $M_{i}$ that differs from $M_{0}$ only in the immediate reward received when action *a* = 1 is taken inside *C*_*i*_. Let


$$b\left(s\right)={\left[1-4L||s-x_{i}{||}_{\infty }/\varepsilon \right]}_{+},$$

where [*z*]_+_ = max{0, *z*}. The function *b*(·) is *L*-Lipschitz, equals 1 at the center *x*_*i*_ and vanishes outside *C*_*i*_. Define


$$r_{i}\left(s,1\right)=\left(1-\gamma \right)\varepsilon b\left(s\right),\;r_{i}\left(s,0\right)=0.$$

The reward amplitude (1−γ)ε is chosen so that the difference in *state values* reaches ε but the instantaneous gap remains small, which will force many samples. Because $|b\left(s\right)-b\left(s^{\prime }\right)|\leq L||s-s^{\prime }{||}_{\infty }$, the pair (*r*_*i*_, *P*_0_) satisfies the global *L*-Lipschitz condition, so each $M_{i}$ lies in M(L).

##### 3.4.1.4 Value functions are well separated

For any *s* ∈ *C*_*i*_, always choosing *a* = 1 gives an immediate reward (1−γ)ε and the agent stays in *s* for ever, so $V_{i}^{⋆}\left(s\right)=\varepsilon$. Outside *C*_*i*_ the two MDPs $M_{i}$ and $M_{0}$ coincide, hence $V_{i}^{⋆}\left(s\right)=0$ there. Consequently


$$||V_{i}^{⋆}-V_{0}^{⋆}{||}_{\infty }=\varepsilon ,\;i\in \left[N\right].$$

The same reasoning shows that $||V_{i}^{⋆}-V_{j}^{⋆}{||}_{\infty }=\varepsilon$ whenever *i*≠*j* because their bumps live in disjoint cubes.

##### 3.4.1.5 Accurate estimation implies correct identification

Suppose an algorithm outputs $\hat{V}$ such that $||\hat{V}-V_{i}^{⋆}{||}_{\infty }\leq \varepsilon /2$. Then necessarily $||\hat{V}-V_{j}^{⋆}{||}_{\infty }\geq \varepsilon /2$ for every *j*≠*i*, hence the algorithm *reveals* the index *i* of the true MDP. Producing an ε-accurate value estimate therefore constitutes solving an *N*-way hypothesis-testing problem.

##### 3.4.1.6 Information each transition conveys

During an episode, the only randomness comes from the reward in the bump region; the state is fixed because the dynamics is a self-loop. Conditional on visiting *C*_*i*_ and picking *a* = 1, the reward is Bernoulli with mean (1−γ)ε under $M_{i}$ and 0 under all $M_{j}$, *j*≠*i*. The KL divergence between these two Bernoulli distributions is upper-bounded by $D_{\text{KL}}\leq 2{\left(1-\gamma \right)}^{2}\varepsilon^{2}$. Thus, each such transition contributes at most a constant multiple of (1−γ)^2^ε^2^ nats of information about the hidden index.

##### 3.4.1.7 Apply Fano's inequality

Let *I*(*i*; *Z*^*T*^) be the mutual information between the hidden index *i* ∈ [*N*] (uniform prior) and the transcript *Z*^*T*^ of the algorithm's first *T* interactions. Because every transition supplies *O*((1−γ)^2^ε^2^) information nats,


$$I\left(i;Z^{T}\right)=O\left(T{\left(1-\gamma \right)}^{2}\varepsilon^{2}\right).$$

Fano's inequality ( Cover and Thomas, 2006, Theorem 2.10.1) gives


$$\mathrm{Pr}\left\{\text{mis-identify}i\right\}\geq 1-\frac{I\left(i;Z^{T}\right)+\log2}{\log N}.$$

To make this error probability at most δ, one must have


$$I\left(i;Z^{T}\right)\geq \frac{1}{2}\log N-\log\frac{1}{\delta }=\text{Ω}\left(\varepsilon^{-d}+\log\frac{1}{\delta }\right).$$

Combining with the upper bound on *I*(*i*; *Z*^*T*^) yields


$$T=\text{Ω}\left({\left(1-\gamma \right)}^{-2}\varepsilon^{-d-2}\log\frac{1}{\delta }\right).$$

This argument is intended to highlight the main curse-of-dimensionality effect: the number of distinguishable regions in a *d*-dimensional state space scales like ε^−*d*^. Thus, aside from problem-dependent factors involving the discount, noise model, and confidence level, the sample requirement takes a representative form.


$$T=\text{Ω}\left({\left(1-\gamma \right)}^{-1}\varepsilon^{-d}\log\left(1/\delta \right)\right).$$

We use this expression as a tutorial summary rather than as a newly derived sharp minimax theorem.

##### 3.4.1.8 Conclusion

Therefore, any algorithm that is uniformly ε-accurate with confidence 1−δ over the class M(L) must, on *some* Lipschitz MDP, experience at least


$$T=\text{Ω}\left({\left(1-\gamma \right)}^{-1}\varepsilon^{-d}\log\frac{1}{\delta }\right)$$

environment interactions in expectation.

Representative Bound 3.4.1 illustrates that even in smooth settings—where both the reward function and transition dynamics vary gradually with the state—RL can still suffer from the curse of dimensionality. In particular, standard minimax lower-bound arguments imply that guaranteeing, with confidence 1−δ, a uniformly accurate value estimate within ε requires on the order of (1−γ)^−1^ε^−*d*^log(1/δ) samples in the worst case. Thus, sample complexity grows exponentially with the state dimension *d*, increases as the discount factor approaches one, and depends logarithmically on the confidence level.

#### 3.4.2 Mitigation strategies

To combat the curse of dimensionality, hierarchical reinforcement learning (HRL) breaks down complex tasks into smaller, manageable subproblems, significantly reducing the complexity of the learning process (Barto and Mahadevan, 2003). Representation learning, such as using autoencoders, can also compress high-dimensional inputs into low-dimensional representations, allowing agents to focus on the most relevant features. Finally, curiosity-driven exploration encourages agents to actively seek out novel states, helping them overcome reward sparsity and improve learning efficiency (Pathak et al., 2017).

From Dimensionality to Efficient Exploration **Problem class**. MDPs with large or continuous state spaces, where naive exploration scales with |S|.

**Canonical limitation**. Sample complexity grows exponentially with dimension in the absence of structure (the curse of dimensionality).

**Representative bound**. Minimax sample complexity scales as

$$\text{Ω}\left(\varepsilon^{-d}\right)$$

for *d*-dimensional state spaces without regularity assumptions.

**Mechanism enabling improvement**. Function approximation, representation learning, and model-based planning reduce dependence on ambient dimension.

**Structural assumption required**. Low intrinsic dimension, Lipschitz continuity, or linear realizability. Under these conditions, complexity depends on effective dimension rather than raw state size.

Representation learning does not eliminate the curse of dimensionality in general. It can reduce effective complexity only when the environment has exploitable structure, such as low intrinsic dimension, smooth dynamics, sparse rewards with reusable features, or approximately linear latent dynamics. Without such assumptions, high-dimensional exploration remains statistically hard even with deep neural networks.

### 3.5 Conditional complexity beyond isolated limits

The following section is conceptual. It does not claim new minimax lower bounds. Instead, it summarizes plausible interaction patterns between known RL difficulties and explains how additional structural assumptions can change effective complexity.

#### 3.5.1 Note on numbering

The results presented here are conceptual propositions rather than formal theorems, and are intended to synthesize known mechanisms rather than introduce new tight bounds.

The preceding sections establish lower bounds for individual obstacles in RL in isolation. In practice, however, multiple challenges often co-occur, and real environments frequently exhibit additional structure that can substantially alter worst-case complexity. This section, therefore, synthesizes three *conditional extensions* that illustrate how statistical difficulty depends on interacting phenomena and structural assumptions rather than on any single factor alone: (i) a compounding (multiplicative) penalty when partial observability and nonstationarity coexist, (ii) a structure-dependent regime in which memory requirements collapse from linear in the horizon to polylogarithmic, and (iii) a probabilistic notion of shielding that yields tunable risk–performance guarantees.

##### 3.5.1.1 Scope and framing

The results in this section are presented as conceptual propositions rather than formal theorems. They synthesize known lower-bound mechanisms and safety arguments to clarify how statistical complexity scales when multiple challenges interact, rather than introducing new proof techniques or claiming tight bounds.

###### 3.5.1.1.1 Notation

For clarity, we use *H* to denote the episode horizon, *K* the number of within-episode change points (piecewise-stationary segments), ε a target performance tolerance, and δ a generic failure probability.

#### 3.5.2 Compounding limits in partially observable nonstationary environments

##### 3.5.2.1 PON-MDP model

We consider episodic environments that are both partially observable and piecewise stationary within an episode.

**Definition 4 [Partially Observable Nonstationary MDP (PON-MDP)]**. A PON-MDP is a tuple $\left(S,A,O,{\left\{P_{k}\right\}}_{k=1}^{K},{\left\{{\text{Ω}}_{k}\right\}}_{k=1}^{K},R,H\right)$, where S,A,O are finite state/action/observation spaces, R:S×A→[0,1] is the reward function, and the transition/emission mechanisms (*P*_*k*_, Ω_*k*_) may switch at most *K* times per episode at unknown times.

##### 3.5.2.2 Synergistic (multiplicative) lower bound

In this compound setting, adaptation to *K* segments and inference under partial observability cannot generally be treated as separable modules: the sample requirement grows multiplicatively rather than additively.

##### 3.5.2.3 Conceptual proposition: synergistic complexity in PON-MDPs

When partial observability and within-episode nonstationarity co-occur in an episodic PON-MDP, learning difficulty can compound rather than add. In particular, for algorithms that aim to return an ε-optimal policy within each stationary segment with high probability, worst-case instances may require several interactions that scales multiplicatively with both the number of change points and the intrinsic difficulty of partial observability.

Heuristically, if partial observability alone induces a difficulty term Ψ(*H*), for example, growing at least linearly with the horizon for finite-memory agents and more rapidly in high-dimensional latent spaces, and nonstationarity alone induces *K* adaptation phases, then the combined regime can exhibit a scaling on the order of *K*·Ψ(*H*). This interaction effect highlights why treating memory management and adaptation as separable algorithmic components can be insufficient in complex environments.

##### 3.5.2.4 Visual summary

**Figure 4:**
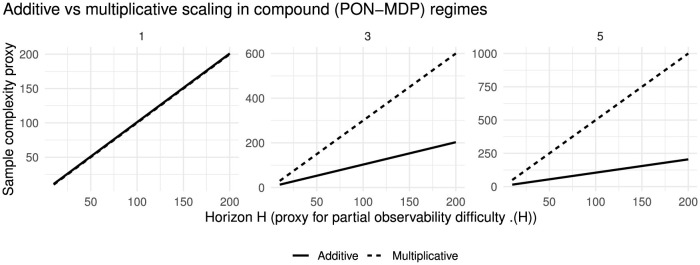
Additive vs. possible multiplicative interaction between difficulties. In PON-MDPs, partial observability and within-episode nonstationarity can compound, yielding *K*·Ψ(*H*) rather than *K*+Ψ(*H*).

Figure 4 contrasts additive versus multiplicative scaling, highlighting the synergistic penalty in the combined regime.

#### 3.5.3 When structure collapses memory requirements

Worst-case POMDP constructions require Θ(*H*) bits of memory for optimality. However, many deployed systems exhibit structured observation models (e.g., low-rank emissions, sparse latent dynamics), enabling substantial compression.

##### 3.5.3.1 Observation (structure-dependent reduction in memory requirements)

Worst-case POMDP constructions require Θ(*H*) bits of memory for optimal control. However, many deployed systems exhibit structured observation models, such as low-rank emission matrices or dynamics confined to a low-dimensional latent subspace. In these settings, belief states can often be represented using compressed sufficient statistics whose dimension depends on intrinsic structural properties rather than on the episode horizon.

As a result, memory requirements may grow only polylogarithmically with the horizon, in sharp contrast to adversarial constructions. This behavior is well documented in work on predictive state representations, spectral methods for POMDPs, and linear dynamical systems under partial observability. It illustrates how additional structure can dramatically reduce effective memory demands.

##### 3.5.3.2 Summary table

Table 2 places this structured regime next to the worst-case baseline.

**Table 2:** Memory scaling regimes under partial observability.

Setting	Memory requirement	Horizon dependence
Worst-case POMDP	Θ(*H*) bits	Linear
Low-rank emissions (rank *r*)	$\tilde{O}\left(r\log H\right)$ bits	Polylogarithmic

##### 3.5.3.3 Visual intuition

**Figure 5:**
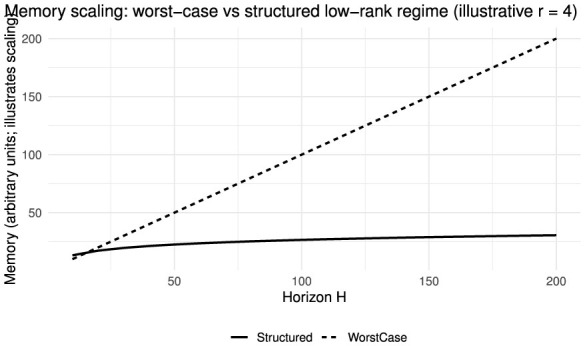
Memory scaling with horizon. Structure (e.g., rank-*r* emissions) collapses the worst-case Θ(*H*) growth to $\tilde{O}\left(r\log H\right)$.

Figure 5 illustrates the horizon scaling gap between Θ(*H*) and $\tilde{O}\left(r\log H\right)$.

#### 3.5.4 Probabilistic safety shielding: a tunable risk–performance dial

Classical safe RL often assumes a *hard* shield that enforces almost-sure safety. In stochastic or uncertain domains, a more realistic aim is to trade off performance and risk via *probabilistic* constraints.

**Definition 5 [Probabilistic shield]**. A probabilistic shield Γ maps each safe state *s* ∈ *S*_safe_ to a distribution Γ(·∣s)∈Δ(A) over actions.

Define the shield quality parameter


$$\alpha :=\underset{s\in S_{\text{safe}}}{\min}\;\underset{a:\;P\left(s^{\prime }\notin S_{\text{safe}}|s,a\right)=0}{\sum }\Gamma \left(a|s\right),$$

i.e. the minimum probability mass that Γ assigns to *guaranteed-safe* actions.

##### 3.5.4.1 Proposition: probabilistic shielding as a risk–performance trade-off

Let π be any base policy. Consider the mixed action rule that, at each step, follows the shield with probability β ∈ (0, 1] and otherwise follows π. Then the probability of staying in *S*_safe_ for at least *T* steps admits a lower bound of the form


$$\mathrm{Pr}(\text{safe for }T\text{ steps})\;\geq \;{\left(\beta \alpha +(1-\beta )\sigma \right)}^{T},$$

where $\sigma :=\underset{s\in S_{\text{safe}},a\in A}{\min}P\left(s^{\prime }\in S_{\text{safe}}|s,a\right)$ is a worst-case one-step safety probability under arbitrary actions. In particular, for pure shielding (β = 1), Pr(safe for*T*steps)≥α^*T*^.

##### 3.5.4.2 Visual summary

**Figure 6:**
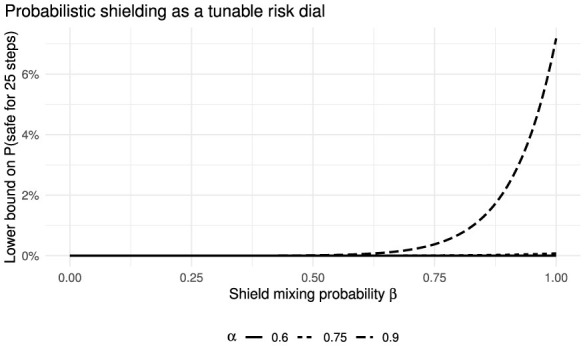
Probabilistic shielding as a tunable dial. Curves show the lower bound (βα+(1−β)σ)^*T*^ vs. β for representative α values.

Figure 6 visualizes the guarantee as a risk dial: increasing β monotonically improves safety, with the curve governed by α.

#### 3.5.5 Implications for conditional complexity

Together, these results motivate a shift from purely worst-case analysis to *conditional complexity*: bounds that tighten when structure is present (e.g. low rank) and that explicitly account for compounding obstacles (e.g. PON-MDPs). Practically, this supports (i) co-designing memory, exploration, and change-point adaptation, (ii) learning or detecting structural rank online to compress policies, and (iii) deploying safety controls via tunable, probabilistic shields that match mission-specific risk budgets.

From Robustness Constraints to Safe Learning **Problem class**. MDPs with model uncertainty or safety constraints that restrict allowable state-action trajectories.

**Canonical limitation**. Optimistic exploration may violate safety constraints or perform poorly under misspecification.

**Representative bound**. Worst-case regret under model uncertainty scales with the size of the uncertainty set and cannot vanish uniformly.

**Mechanism enabling improvement**. Robust MDP formulations, minimax optimization, and risk-sensitive objectives such as CVaR.

**Structural assumption required**. Known uncertainty sets, convexity, or conservative baseline policies. These enable safety guarantees at the cost of reduced asymptotic optimality.

## 4 Emerging approaches and ongoing research

### 4.1 Safe and secure RL

In safety-critical domains, such as autonomous driving or healthcare, ensuring the safe and secure operation of RL agents is paramount. Constrained or shielded exploration techniques have been developed to prevent agents from taking actions that could lead to undesirable or hazardous outcomes (Amodei et al., 2016). These methods incorporate safety constraints into the learning process, ensuring that exploration adheres to predefined safety boundaries. By prioritizing safety during training and deployment, these approaches aim to make RL more reliable in real-world applications where errors could have severe consequences.

### 4.2 Explainable RL

As RL systems are increasingly deployed in high-stakes domains such as healthcare and finance, interpretability has become an important practical consideration (Puiutta and Veith, 2020; Jaques et al., 2019). Explainable RL aims to improve transparency and trust through methods such as saliency maps, which identify influential input features, and policy distillation, which simplifies complex policies into more interpretable forms. These approaches support debugging, accountability, and alignment with ethical and operational requirements. Supplementary Figure S2 illustrates the role of explainable RL in high-stakes environments.

### 4.3 Multi-agent RL

Multi-agent reinforcement learning (MARL) considers settings involving multiple interacting agents, including drone fleets, multi-robot coordination, and competitive games (Lowe et al., 2017; Jaques et al., 2019). These settings introduce additional challenges, including emergent behavior, inter-agent credit assignment, communication, and scalability as the joint state-action space grows. Current MARL research focuses on coordination mechanisms and scalable learning strategies for deployment in dynamic multi-agent environments. Supplementary Figure S3 summarizes applications, challenges, and solution strategies in MARL.

### 4.4 Automatic curriculum learning

Automatic Curriculum Learning (ACL) is a promising approach that improves sample efficiency and robustness in RL by organizing tasks into a structured progression of increasing difficulty. Instead of exposing agents to complex tasks from the outset, ACL introduces simpler tasks first, allowing agents to gradually build the skills necessary to tackle more challenging problems. This method mirrors how humans and animals learn, progressively mastering foundational skills before advancing to more complex ones.

Florensa et al. (2018) demonstrated the effectiveness of automatic curriculum generation, showing that agents trained with a curriculum can achieve performance gains compared with agents trained without such a structure. By dynamically adjusting the difficulty of tasks based on the agent's current capabilities, ACL ensures that learning remains challenging yet achievable, minimizing wasted effort on tasks that are either too easy or overly difficult.

This approach not only accelerates learning but also enhances robustness by exposing agents to a diverse range of tasks and scenarios. As a result, ACL is becoming an essential tool in RL, particularly in environments with high-dimensional state spaces or sparse rewards. Ongoing research is exploring ways to generalize automatic curriculum learning to more complex, real-world applications, further unlocking its potential for scalable and efficient training.

## 5 Conclusions

This survey has provided a tutorial synthesis of four statistical challenges that limit the deployment of RL in real-world settings: sample inefficiency, nonstationarity, partial observability, and high dimensionality. Rather than treating these issues as isolated problems, the paper organized them around their associated statistical limits, representative lower bounds, and structural assumptions that can reduce effective complexity.

The main contribution of the survey is the conditional-complexity perspective: real-world RL difficulty depends not only on worst-case sample complexity, but also on the interaction between memory, adaptation, safety, and representation structure. This perspective helps explain why certain methods succeed under strong assumptions but remain fragile when these assumptions fail.

The paper does not claim new formal lower bounds. Its contribution is instead organizational and interpretive: it clarifies how existing theoretical results and practical mitigation strategies fit into a unified framework.

The synthetic simulation and conceptual conditional-complexity examples are therefore used only for illustration and for organizing hypotheses, not for empirical validation or as standalone theoretical contributions.

By bringing together results and intuitions scattered across the reinforcement learning, control, and information-theoretic literatures, this paper clarifies three recurring themes. First, when partial observability and non-stationarity co-occur, learning difficulty can compound rather than add, underscoring the limitations of modular, patch-by-patch algorithm design. Second, the presence of exploitable structure, such as low-rank observation models or low-dimensional latent dynamics, can dramatically relax worst-case memory requirements, legitimizing model compression and resource-aware deployment in realistic settings. Third, probabilistic notions of safety provide a principled way to navigate the trade-off between performance and risk, bridging the gap between brittle never-fail constraints and the stochastic nature of real environments.

## Data Availability

The original contributions presented in the study are included in the article/Supplementary material, further inquiries can be directed to the corresponding author.
