# Supplementary material for: Statistical limits and conditional complexity in real-world reinforcement learning: a tutorial survey
Source: Front Artif Intell. 2026 Jun 24;9:1847643. doi: 10.3389/frai.2026.1847643 (PMC13341602; doi:10.3389/frai.2026.1847643)
Supplement: Supplementary file 1 [file Supplementary_file_1.pdf]

Supplementary Information

Statistical Limits and Conditional Complexity in Real-World Reinforcement Learning: A Tutorial Survey

Amar Ahmad, Yvonne Vallès, and Youssef Idaghdour

This supplementary material accompanies the article:

Statistical Limits and Conditional Complexity in Real-World Reinforcement Learning: A Tutorial Survey

Supplementary Information

Scope and intent. This supplementary material provides additional conceptual clarification and interpretive context for selected discussions in the main text. It supports the tutorial–survey nature of the article by expanding on intuition and qualitative behaviour, rather than introducing new theoretical results.

Important note. The arguments presented here are not claimed as novel theorems, formal lower bounds, or original proofs. Instead, they synthesise and reinterpret well-established ideas from reinforcement learning, information theory, and control to explain why particular scaling regimes and trade-offs arise when multiple challenges interact. *Formal proofs are deliberately omitted.*

S1. Synergistic Difficulty Under Combined Challenges

Relation to main text. This section supports the discussion in Section 3.5 on the interaction between partial observability and nonstationarity.

Key intuition. Lower bounds for partially observable environments typically arise because an agent must infer latent state information from histories whose effective length scales with the episode horizon. Separately, nonstationary (or piecewise-stationary) environments require repeated adaptation as dynamics change.

When these challenges occur simultaneously, inference cannot be amortised across time: each stationary segment effectively induces a *new* latent-state identification problem. As a result, learning difficulty can compound across segments rather than adding linearly.

Interpretation. This phenomenon should be understood as a worst-case interaction effect. It highlights why algorithmic components such as memory, exploration, and adaptation cannot always be designed independently in complex environments. No claim of tightness or novelty is made.

S2. Structure and Memory Compression

Relation to main text. This section provides additional intuition for the discussion of structured observation models in Section 3.5.

Key intuition. Worst-case memory requirements in partially observable decision processes are driven by adversarial constructions in which each observation conveys an independent piece of information that must be retained until acted upon. In contrast, many practical environments exhibit exploitable structure (e.g., low-rank observation models or dynamics confined to a low-dimensional latent subspace).

In such settings, belief states admit compressed representations, and memory requirements can grow logarithmically with the horizon rather than linearly. This behaviour is well documented in work on predictive state representations, spectral methods, and linear dynamical systems.

Interpretation. The discussion in the main text highlights a structure-dependent regime rather than proposing a new memory bound.

S3. Probabilistic Safety as a Risk–Performance Trade-off

Relation to main text. This section supports the discussion of probabilistic safety mechanisms in Section 3.5.

Key intuition. Hard safety constraints can enforce zero risk but may severely limit exploration and performance. Probabilistic safety mechanisms instead mix a base policy with a safety controller, allowing risk to be tuned explicitly.

At each decision step, safety is preserved with some minimum probability determined by (i) the quality of the safety mechanism and (ii) the degree of intervention. Over multiple steps, these probabilities compound multiplicatively, yielding an exponential dependence on the time horizon.

Interpretation. The value of this perspective lies in making explicit the trade-off between safety and autonomy. The resulting guarantees follow from elementary probability and are not claimed as new theoretical results.

S4. Scope and Limitations

The supplementary arguments above are intended solely to clarify intuition, unify related concepts under a conditional-complexity perspective, and guide algorithmic thinking. They are not claimed to be tight, exhaustive, or novel. Any rigorous formalisation would require additional problem-specific assumptions and lies beyond the scope of this survey.

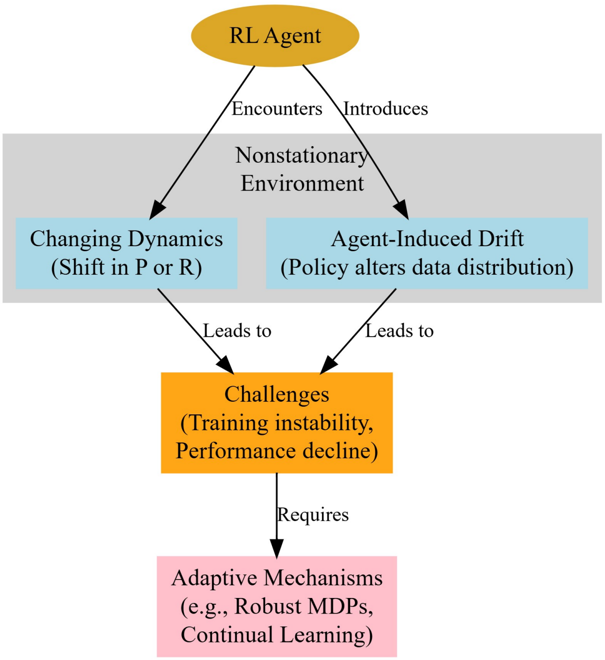

Figure S1: Challenges faced by reinforcement learning agents in nonstationary environments.

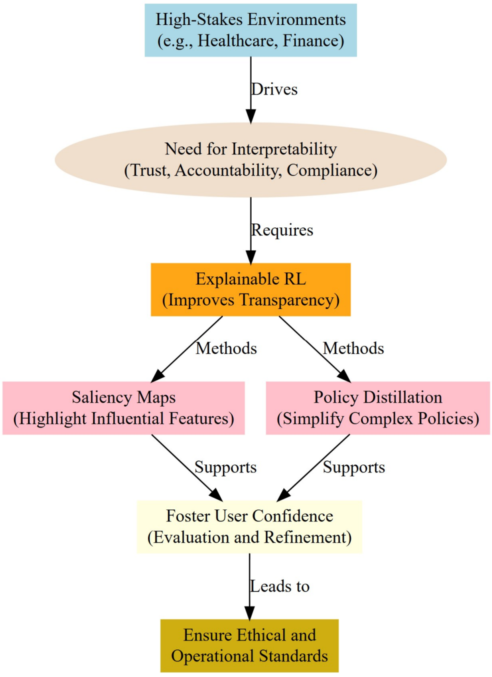

Figure S2: The role of explainable reinforcement learning (RL) in high-stakes environments. The diagram highlights the need for interpretability to foster trust, accountability, and compliance. Key methods such as saliency maps and policy distillation improve transparency, enabling stakeholders to evaluate and refine RL systems while ensuring alignment with ethical and operational standards.

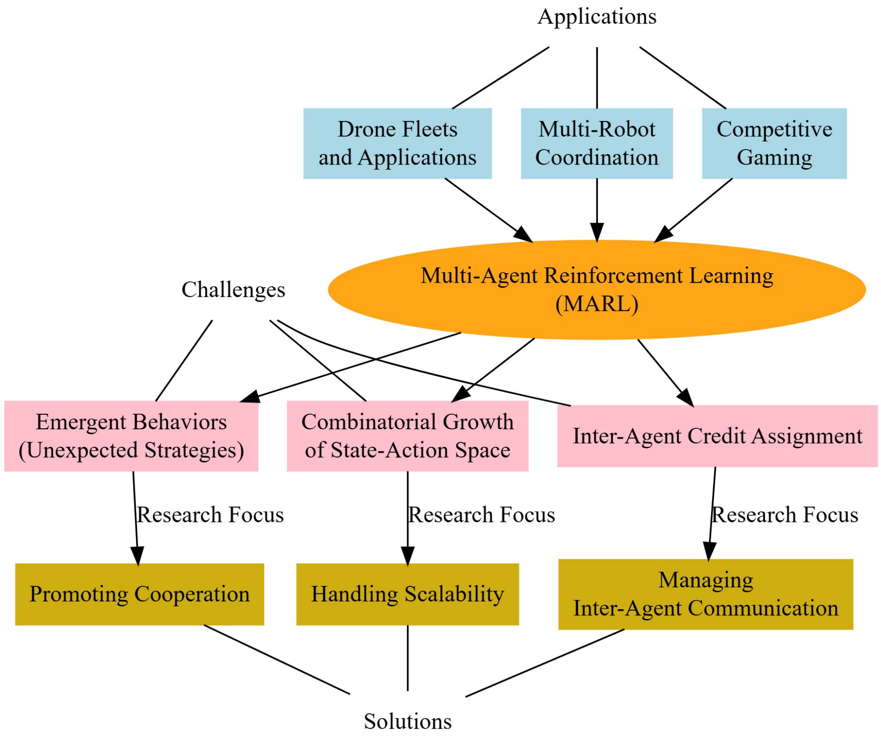

Figure S3: A high-level framework for multi-agent reinforcement learning (MARL), illustrating representative applications, core challenges, and solution strategies.
