# Supplementary material for: Statistical limits and conditional complexity in real-world reinforcement learning: a tutorial survey
Source: Front Artif Intell. 2026 Jun 24;9:1847643. doi: 10.3389/frai.2026.1847643 (PMC13341602; doi:10.3389/frai.2026.1847643)
Supplement: Supplementary file 2 [file Supplementary_file_2.docx]

\documentclass[12pt]{article}

\usepackage[a4paper,margin=1in]{geometry}

\usepackage{graphicx}

\usepackage{amsmath,amssymb}

\usepackage{hyperref}

\setcounter{figure}{0}

\setcounter{table}{0}

\renewcommand{\thefigure}{S\arabic{figure}}

\renewcommand{\thetable}{S\arabic{table}}

\begin{document}

\begin{center}

{\LARGE \textbf{Supplementary Information}}\\[1.5em]

{\large

\textbf{Statistical Limits and Conditional Complexity in Real-World

Reinforcement Learning: A Tutorial Survey}

}\\[1em]

Amar Ahmad, Yvonne Vall\`es, and Youssef Idaghdour

\end{center}

\vspace{1cm}

\noindent

This supplementary material accompanies the article:

\medskip

\noindent

{\large

\textbf{Statistical Limits and Conditional Complexity in Real-World

Reinforcement Learning: A Tutorial Survey}

}

\vspace{1cm}

%========================

% Supplementary Information

%========================

\section*{Supplementary Information}

\addcontentsline{toc}{section}{Supplementary Information}

\paragraph{Scope and intent.}

This supplementary material provides additional conceptual clarification and

interpretive context for selected discussions in the main text. It supports the

tutorial--survey nature of the article by expanding on intuition and qualitative

behaviour, rather than introducing new theoretical results.

\paragraph{Important note.}

The arguments presented here are not claimed as novel theorems, formal lower bounds,

or original proofs. Instead, they synthesise and reinterpret well-established ideas

from reinforcement learning, information theory, and control to explain why particular

scaling regimes and trade-offs arise when multiple challenges interact.

\emph{Formal proofs are deliberately omitted.}

%------------------------

\subsection*{S1. Synergistic Difficulty Under Combined Challenges}

\paragraph{Relation to main text.}

This section supports the discussion in Section~3.5 on the interaction between

partial observability and nonstationarity.

\paragraph{Key intuition.}

Lower bounds for partially observable environments typically arise because an agent

must infer latent state information from histories whose effective length scales with

the episode horizon. Separately, nonstationary (or piecewise-stationary) environments

require repeated adaptation as dynamics change.

When these challenges occur simultaneously, inference cannot be amortised across time:

each stationary segment effectively induces a \emph{new} latent-state identification

problem. As a result, learning difficulty can compound across segments rather than

adding linearly.

\paragraph{Interpretation.}

This phenomenon should be understood as a worst-case interaction effect. It highlights

why algorithmic components such as memory, exploration, and adaptation cannot always be

designed independently in complex environments. No claim of tightness or novelty is made.

%------------------------

\subsection*{S2. Structure and Memory Compression}

\paragraph{Relation to main text.}

This section provides additional intuition for the discussion of structured observation

models in Section~3.5.

\paragraph{Key intuition.}

Worst-case memory requirements in partially observable decision processes are driven by

adversarial constructions in which each observation conveys an independent piece of

information that must be retained until acted upon. In contrast, many practical

environments exhibit exploitable structure (e.g., low-rank observation models or

dynamics confined to a low-dimensional latent subspace).

In such settings, belief states admit compressed representations, and memory requirements

can grow logarithmically with the horizon rather than linearly. This behaviour is well

documented in work on predictive state representations, spectral methods, and linear

dynamical systems.

\paragraph{Interpretation.}

The discussion in the main text highlights a structure-dependent regime rather than

proposing a new memory bound.

%------------------------

\subsection*{S3. Probabilistic Safety as a Risk--Performance Trade-off}

\paragraph{Relation to main text.}

This section supports the discussion of probabilistic safety mechanisms in Section~3.5.

\paragraph{Key intuition.}

Hard safety constraints can enforce zero risk but may severely limit exploration and

performance. Probabilistic safety mechanisms instead mix a base policy with a safety

controller, allowing risk to be tuned explicitly.

At each decision step, safety is preserved with some minimum probability determined by

(i) the quality of the safety mechanism and (ii) the degree of intervention. Over multiple

steps, these probabilities compound multiplicatively, yielding an exponential dependence

on the time horizon.

\paragraph{Interpretation.}

The value of this perspective lies in making explicit the trade-off between safety and

autonomy. The resulting guarantees follow from elementary probability and are not claimed

as new theoretical results.

%====================================================

% Supplementary Figures

%====================================================

%====================================================

% Supplementary Figures

%====================================================

\begin{figure}[ht]

\centering

\includegraphics[width=.72\textwidth]{ChallengesNonstatEnviroInRL.pdf}

\caption{Challenges faced by reinforcement learning agents in nonstationary environments.}

\label{fig:supp-nonstationarity}

\end{figure}

\begin{figure}[ht]

\centering

\includegraphics[width=.75\textwidth]{RoleExplainableRLinHighStakesEnvir.pdf}

\caption{The role of explainable reinforcement learning (RL) in high-stakes environments. The diagram highlights the need for interpretability to foster trust, accountability, and compliance. Key methods such as saliency maps and policy distillation improve transparency, enabling stakeholders to evaluate and refine RL systems while ensuring alignment with ethical and operational standards.}

\label{fig:supp-explainableRL}

\end{figure}

\begin{figure}[ht]

\centering

\includegraphics[width=.80\textwidth]{MARLapplicationsChallengesSolutionsDiagram.pdf}

\caption{A high-level framework for multi-agent reinforcement learning (MARL), illustrating representative applications, core challenges, and solution strategies.}

\label{fig:supp-marl}

\end{figure}

%------------------------

\subsection*{S4. Scope and Limitations}

The supplementary arguments above are intended solely to clarify intuition, unify related

concepts under a conditional-complexity perspective, and guide algorithmic thinking.

They are not claimed to be tight, exhaustive, or novel. Any rigorous formalisation would

require additional problem-specific assumptions and lies beyond the scope of this survey.

\end{document}
